# Incorporating variable RBE in IMPT optimization for ependymoma

**DOI:** 10.1002/acm2.14207

**Published:** 2023-11-20

**Authors:** Hadis Moazami Goudarzi, Gino Lim, David Grosshans, Radhe Mohan, Wenhua Cao

**Affiliations:** ^1^ Department of Industrial Engineering University of Houston Houston Texas USA; ^2^ Department of Radiation Oncology The University of Texas MD Anderson Cancer Center Houston Texas USA; ^3^ Department of Radiation Physics The University of Texas MD Anderson Cancer Center Houston Texas USA

**Keywords:** ependymoma, IMPT, LET, optimization, RBE

## Abstract

**Purpose:**

To study the dosimetric impact of incorporating variable relative biological effectiveness (RBE) of protons in optimizing intensity‐modulated proton therapy (IMPT) treatment plans and to compare it with conventional constant RBE optimization and linear energy transfer (LET)‐based optimization.

**Methods:**

This study included 10 pediatric ependymoma patients with challenging anatomical features for treatment planning. Four plans were generated for each patient according to different optimization strategies: (1) constant RBE optimization (ConstRBEopt) considering standard‐of‐care dose requirements; (2) LET optimization (LETopt) using a composite cost function simultaneously optimizing dose‐averaged LET (LET_d_) and dose; (3) variable RBE optimization (VarRBEopt) using a recent phenomenological RBE model developed by McNamara et al.; and (4) hybrid RBE optimization (hRBEopt) assuming constant RBE for the target and variable RBE for organs at risk. By normalizing each plan to obtain the same target coverage in either constant or variable RBE, we compared dose, LET_d_, LET‐weighted dose, and equivalent uniform dose between the different optimization approaches.

**Results:**

We found that the LETopt plans consistently achieved increased LET in tumor targets and similar or decreased LET in critical organs compared to other plans. On average, the VarRBEopt plans achieved lower mean and maximum doses with both constant and variable RBE in the brainstem and spinal cord for all 10 patients. To compensate for the underdosing of targets with 1.1 RBE for the VarRBEopt plans, the hRBEopt plans achieved higher physical dose in targets and reduced mean and especially maximum variable RBE doses compared to the ConstRBEopt and LETopt plans.

**Conclusion:**

We demonstrated the feasibility of directly incorporating variable RBE models in IMPT optimization. A hybrid RBE optimization strategy showed potential for clinical implementation by maintaining all current dose limits and reducing the incidence of high RBE in critical normal tissues in ependymoma patients.

## 1 INTRODUCTION

Proton therapy has become an increasingly important treatment option for cancer patients because of its dosimetrical advantages over photon therapy.^1^ Protons' physical characteristics make it possible to generate highly conformal radiation treatment plans in which the desired dose surrounds the target tightly while the dose deposited in nearby normal tissues is minimized. In the current practice of proton beam therapy, prescribed doses are usually determined by scaling the physical proton dose using a proton relative biological effectiveness (RBE) value of 1.1.^2^ The constant RBE value of 1.1 is based on the assumption that protons are 10% more biologically effective than photons and accounts for this higher cell‐killing efficiency regardless of tissue type. However, in vitro and in vivo studies indicate that RBE varies within a treatment field according to physical and biological factors.^3^ Proton therapy might be less effective if such variability is ignored. Because of the assumption of constant RBE, coupled with physical uncertainties during treatment delivery, the distribution of “true” biologically effective doses received by the patient may differ from what is indicated on the treatment plan to an unknown, possibly significant, magnitude. Consequently, unanticipated toxicities may occur and/or the patient's disease may not be controlled.^4^, ^5^, ^6^, ^7^

While the impact of physical uncertainties involved in proton therapy, such as patient setup and proton range uncertainties, may be effectively mitigated by robust optimization techniques, which are increasingly being implemented in clinical practice, RBE variations and appropriate treatment planning strategies are still under active investigation. RBE is known to depend on particle type, linear energy transfer (LET), tissue type (α/β ratio), dose per fraction, and biological endpoint, among other factors.^8^, ^9^, ^10^, ^11^ Various RBE models have been developed to calculate biologically effective (RBE‐weighted) dose distributions using in vitro cell survival data for particles such as carbon ions and protons. The local effect model is a prominent RBE model for carbon ions and has been applied clinically in carbon ion therapy.^12^, ^13^ Although currently only constant RBE is used in clinical proton therapy as the standard for dose prescription and treatment planning, clinical proton centers are actively investigating the risks of RBE variations and evaluating RBE‐weighted dose using LET–parameterized RBE models to various extents.^14^ These models include either empirical or mechanism‐based ones, which are all established by applying the linear‐quadratic model with radiation sensitivity parameters α andβ for protons.^15^, ^16^, ^17^, ^18^, ^19^, ^20^ Frese et al. first studied the feasibility of including variable RBE in intensity‐modulated proton therapy (IMPT) optimization using a cost function defined by biological effect, that is, $\alpha D+\beta D^{2}$ (*D* is absorbed dose and parameters α andβ are for protons).^18^, ^21^

In contrast to anticipated dependence of RBE on LET among other factors, there are also studies suggesting that RBE may not correlate with LET clinically.^22^, ^23^ For example, Niemierko et al. analysis on 50 brains cases suggests despite the increase in LET and RBE towards the end of the range, the actual impact on patients may be relatively modest in comparison to the inherent interpatient variability in radiosensitivity.^22^

Nevertheless, current proton RBE models are still associated with substantial uncertainties in biological measurement, interpretation of measured assay data, dosimetry, and assumptions about simulated mechanisms. While published studies of in vitro experiments indicate nonnegligible uncertainties in RBE models, they agree that RBE of protons increases linearly with dose‐averaged LET, and non‐linearly at higher LETs near and beyond the Bragg peak. This highlights the prediction that RBE climbs to significantly higher than 1.1 near the end of proton beam range, where LET increases sharply. Therefore, recent research has put extensive attention on incorporating LET instead of RBE into treatment planning, so that the uncertainties in biophysical RBE models are avoided. In addition, LET can be accurately calculated because it is entirely based on physical properties.

LET‐based IMPT optimization can be achieved either by a two‐step approach, in which first physical dose is optimized and second LET is optimized with limited change to physical dose,^24^ or in a simultaneous manner using a composite cost function of both LET and dose.^25^, ^26^ Some other recent studies incorporating LET criteria include proton track‐end optimization,^27^ beam angle optimization,^28^ and robust optimization.^29^ The goal of LET optimization is to increase LET in target regions and decrease it in normal tissues while maintaining the dose constraints (in constant RBE) specified in current practice. However, the obvious drawback of LET optimization is that biologically effective dose does not depend upon LET alone and, thus, increasing or decreasing LET in a tumor or normal tissue would not correctly reflect its clinical consequences.

The main objective of the present study was two‐fold. The first was to assess the impact of variable RBE‐weighted dose optimization on physical dose distribution compared to conventional and LET optimization; the second was to investigate the efficacy of variable RBE optimization in improving LET and RBE effect compared to LET optimization. We focused this study on a cohort of pediatric ependymoma patients, as these cases often present critical serial organs, for example, the brainstem and spinal cord, in very close proximity to the tumorous area; therefore, preventing overdosing or underdosing of biological dose is highly important. In addition, we explored a variant approach of biological optimization in which variable RBE is only considered for critical normal tissues to demonstrate another possible scenario in which RBE models could be incorporated into treatment planning with current clinical prescription protocols.

It should be highlighted that our study does not aim to provide a comprehensive discussion of the clinical implications of variable RBE optimization; instead, it attempts to show some exploratory evidence on how variable RBE can be directly incorporated into treatment planning and the dosimetric effect of doing so, via comparisons to multiple competing approaches.

## 2 MATERIALS AND METHODS

In this study, four IMPT plan optimization approaches, constant RBE optimization (ConstRBEopt), LET‐based optimization (LETopt), variable RBE optimization (VarRBEopt), and hybrid RBE optimization (hRBEopt), were implemented and compared. While variable RBE weighted dose was calculated for optimization in VarRBEopt and hRBEopt, only constant RBE weighted dose was used in LETopt and ConstRBEopt, for plan generation. However, both variable and constant RBE weighted doses for all four plans were calculated for plan evaluation.

The physical dose $D_{i}$ and dose‐averaged LET (LET_d_) $L_{i}$ are calculated as follows:

(1) $$\;D_{i}=\sum_{j}D_{ij}\;w_{j},$$

(2) $$\;L_{i}=\frac{\sum_{j}D_{ij}L_{ij}w_{j}}{\sum_{j}D_{ij}w_{j}},$$

where $D_{ij}\;$and $L_{ij}$ are the dose and LET contributions from beamlet *j* to voxel *i* in unit intensity, respectively, and *w_j_* indicates the intensity of beamlet *j*.

The cost function for the ConstRBEopt, VarRBEopt, and hRBEopt models a sum of quadratic terms penalizing deviations between achieved and prescribed doses, as demonstrated in Equation (3).

(3) $$\begin{array}{ccc} F_{D}\left(w\right) & = & \sum_{i=1}^{N_{T}}\frac{\lambda_{T}^{+}}{\left|N_{T}\right|}{\left(RBE_{i}\cdot D_{i\in T}-D_{i\in T}^{pr}\right)}_{+}^{2}\\ & & +\sum_{i\;=\;1}^{N_{T}}\frac{\lambda_{T}^{-}}{\left|N_{T}\right|}{\left(RBE_{i}\cdot D_{i\in T}-D_{i\in T}^{pr}\right)}_{-}^{2}\\ & & +\sum_{i\;=\;1}^{N_{O}}\frac{\lambda_{O}^{+}}{\left|N_{O}\right|}{\left(RBE_{i}\cdot D_{i\in O}-D_{i\in O}^{pr}\right)}_{+}^{2} \end{array}$$

In (3), $D_{i\in T}^{pr}$ and $D_{i\in O}^{pr}$ demonstrate the prescribed dose in Gy(RBE) for tumor and organs at risk (OARs), respectively; $\lambda_{T}^{+}$ and $\lambda_{T}^{-}$ are penalty weighting factors for overdosing and underdosing the target, respectively; $\lambda_{O}^{+}$ is for normal tissue overdosing; $N_{T}$ and $N_{O}\;$are the numbers of target and OAR voxels, respectively. For ConstRBEopt, $RBE_{i}$ is set to 1.1 for all voxels. For VarRBEopt, $RBE_{i}$ is calculated using the model described by McNamara et al.^15^ For hRBEopt, $RBE_{i}$ is constant 1.1 for target voxels and variable (also using the McNamara model) for OAR voxels. When target and OARs overlap, constant RBE is used for those overlapping voxels.

The RBE model introduced by McNamara et al.^15^ is shown in Equation (4). We consider two tissue‐specific parameters, that is, ${\left(\alpha /\beta \right)}_{x}=\;2$ for OARs (brainstem and spinal cord) and ${\left(\alpha /\beta \right)}_{x}=\;10$ for tumor, in this study.

(4) $$\begin{array}{ccc} & & RBE\left[D_{P},\;\frac{\alpha }{\beta },\;LET_{d}\right]\\ & & \;=\frac{1}{2D_{P}}\left(\sqrt{{{\left(\frac{\alpha }{\beta }\right)}_{x}}^{2}+4D_{P}{\left(\frac{\alpha }{\beta }\right)}_{x}\left(0.999064+\frac{0.35605}{{\left(\frac{\alpha }{\beta }\right)}_{x}}LET_{d}\right)+4{D_{P}}^{2}{\left(1.1012-0.0038703\sqrt{{\left(\frac{\alpha }{\beta }\right)}_{x}}\;LET_{d}\right)}^{2}}-{\left(\frac{\alpha }{\beta }\right)}_{x}\right) \end{array}$$

The cost function for LETopt (5) is formulated by adding two quadratic terms, maximizing LET in target and minimizing LET in OARs, to the dose‐only cost function (3). The goal of this cost function is to optimize dose and LET distributions simultaneously.

(5) $$\;F_{L}\;\left(w\right)=F_{D}\;\left(w\right)-\frac{\gamma_{T}}{\left|N_{T}\right|}\sum_{i\;=\;1}^{N_{T}}L_{i\in T}^{2}+\frac{\gamma_{O}}{\left|N_{O}\right|}\sum_{i\;=\;1}^{N_{O}}L_{i\in O}^{2}$$

In (5), $\gamma_{T}$ and $\gamma_{O}$ are weighting factors to control the priorities of LET in target and OARs, respectively.

We used matRad,^30^ an open‐source treatment planning system for radiation therapy written in Matlab, to create all IMPT plans and produce dose and LET influence matrices. The voxel size was set to 3 × 3 × 3 mm^3^. The lateral spot spacing was 5 mm, and the energy layers were interpolated uniformly with a spacing of 2 mm in the beam direction. Analytical models were used in matRad for calculating dose^31^ and LET.^32^ The accuracy of the models was validated by Monte Carlo calculations in previous studies.^32^, ^33^ All the IMPT optimization problems mentioned in this paper are highly non‐convex. Hence, we solved those problems using the Interior Point Optimizer (IPOPT),^34^ a solver developed for large‐scale nonlinear optimization instances and also included in matRad. All computations were performed on a laptop with an Intel Core i7 CPU (3.6 GHz) and 12 GB of RAM.

For all 10 pediatric ependymoma cases, a dose prescription of 54 Gy(RBE) (RBE‐weighted dose) was prescribed for delivery in 30 fractions to the clinical target volume (CTV). The OARs considered were brainstem and spinal cord for all patients. The maximum voxel dose constraints were 50 Gy(RBE) for the spinal cord and 57 Gy(RBE) for the brainstem. Note that we considered variable RBE weighted dose for all dose constraints (target and OARs) for VarRBEopt, and constant RBE weighted dose for LETopt and ConstRBEopt, whereas hRBEopt used constant RBE for target constraints and variable RBE for OAR constraints. The CTV was set as the optimization target. Here, all plans utilized the clinically used treatment field angles for each patient. Details of the beam angles, number of voxels, and number of beamlets for each patient are shown in Table 1.

**TABLE 1:** Patient information and key treatment planning parameters.

Case #	Beam angles (Gantry, Couch)	Number of beamlets	Total number of voxels (Target, Brainstem, Spinal Cord)	Number of overlapping voxels (Target ∩ Brainstem)
1	(100,12), (260,348), (280,45), (80,315)	1248	1100 (661, 385, 54)	88
2	(180,0), (270,15), (90,345)	2578	2047 (1529, 391, 127)	116
3	(245,0), (180,0), (112,0), (315,0)	2044	2099 (907, 719, 473)	87
4	(60,0), (260,15), (305,0), (160,0)	5313	3365 (2688, 372, 305)	284
5	(290,345), (70,15)	1261	2121 (1230, 805, 86)	0
6	(155,0), (75,0), (310,0), (205,0)	1374	1316 (695, 362, 259)	59
7	(325,0), (252,20), (175,0), (100,0)	2001	1482 (995, 436, 51)	27
8	(105,0), (255,0), (285,90)	1018	1514 (680, 739, 95)	75
9	(290,90), (270,0), (90,0)	1110	2229 (801, 1043, 385)	169
10	(290,90), (270,0), (90,0), (180,0)	2096	1854 (1014, 657, 183)	52

It should be mentioned that each of the four plans per patient was optimized independently, with a starting condition of uniform beamlet intensity. No base plan was used to create the LETopt, VarRBEopt, and hRBEopt plans. Furthermore, in plan generation, each plan was normalized to meet the same target coverage, that is, 90% of the CTV covered by the prescribed dose, after optimization. Note that the plan normalization in this study was based on RBE weighted dose with different RBE schemes according to different plans. In other words, the VarRBEopt plan was normalized so that the variable RBE weighted dose reaches the target coverage benchmark, but the other three plans used constant RBE‐weighted dose for normalization. In plan evaluation, all plans were re‐calculated using both constant and variable RBE. For comparison purposes, the VarRBEopt plans were also re‐normalized so that constant RBE weighted dose could meet the target coverage benchmark. The choice of using 90% target coverage of prescription dose as the normalization benchmark is based on our experience in planning these patients for original IMPT treatments in the clinic. Most patients present complex concave tumor shapes and considerable proximity or overlap between target volume and brainstem. We found 90% target coverage (lower than typical clinical protocols) is a reasonable threshold to balance the need to meet the critical organ dose limit (protecting brainstem and spinal cord as top priority for pediatric patients) and increase target dose as much as possible.^35^

To study the effects of the different optimization strategies, various dosimetric measures were evaluated, including distributions of doses recalculated in both constant and variable RBE, dose‐volume histograms (DVHs), dose‐averaged LET (LET_d_), LET‐volume histograms (LEV‐VHs), maximum (and mean) dose and LET_d_ to a voxel, as well as generalized equivalent uniform dose (gEUD).^36^, ^37^ We also included analysis of LET‐weighted dose (c LET × D), a metric embraced in many recent studies.^24^, ^28^, ^29^, ^38^, ^39^ If biological dose or RBE‐weighted dose is defined by a simple LET parameterized form: D + c LET × D, the product of LET and physical dose D (i.e., LET‐weighted dose), can be seen as an extra component of LET effect in total biological dose, which “models” the linear increase of RBE with LET. For single dosimetric indices per structure, such as mean and maximum dose or LET, we used paired *t*‐test to determine if the mean difference between two treatment plans is significantly different than 0, or if the average is significantly different. A confidence level of 95% was chosen for hypothesis testing and *p*‐value ≤ 0.05 was considered statistically significant for a particular plan quality index in a two‐plan comparison.

In addition, we introduced and exploited the differential EUD concept to evaluate the differential gain of biological based optimization compared to conventional optimization. The differential EUD quantifies the EUD difference between a test plan TP (e.g., a LET/VarRBE/hRBE optimization plan) and a reference plan RP (e.g., a ConstRBE optimization plan) for a given volume of interest (VOI), that is, $\Delta EUD_{VOI}$ as defined by Equation (6). The sum of differential EUD can present a single composite score comparing two plans by including multiple VOIs (target volumes and normal tissues), that is, ΔEUD as defined by the linear Equation (7).

(6) $$\Delta EUD_{VOI}\left(TP-RP\right)\;=\;EUD_{VOI}\left(TP\right)-EUD_{VOI}\left(RP\right)$$

(7) $$\Delta EUD\;=\sum_{k}\gamma_{T_{k}}\times \Delta EUD_{T_{k}}\;-\sum_{k}\gamma_{N_{k}}\times \Delta EUD_{N_{k}}$$

When computing $\Delta EUD,\;\gamma_{T_{k}}$ and $\gamma_{N_{k}}$ are user‐defined parameters to balance the priority of each of the target volumes and normal tissues in a specific evaluation. We used equal weights $(\gamma_{T_{k}}=\;1,$ $\gamma_{N_{k}}=\;1)$ in this study.

Here the organ specific EUD is defined by generalized EUD (gEUD) in this study.^36^, ^37^ The gEUD for each VOI is calculated by the following formula,

(8) $$gEUD\;=\;{\left(\sum_{i}v_{i}D_{i}^{a}\right)}^{\frac{1}{a}},\;$$

where $v_{i}$ is representing the fractional organ volume receiving a dose $D_{i}$, and *a* is a tissue‐specific parameter that characterizes the volume effect and varies according to the tissue type. In gEUD calculation, we chose a value of −10 for the parameter *a* for the target to mimic the effect of cold spots on tumor control probability and 10 for the brainstem and spinal cord to reflect the dependence of highest dose in the tissue for serial organs.^40^, ^41^, ^42^ We should note that ΔEUD can be measured for either variable or constant RBE‐weighted dose distributions. A positive value of ΔEUD in the present definition indicates a gain in EUD for the test plan over the reference plan. For a specific VOI, a positive value of $\Delta EUD_{VOI}$ simply means higher EUD of the test plan than the reference plan.

## 3 RESULTS

**FIGURE 1:**
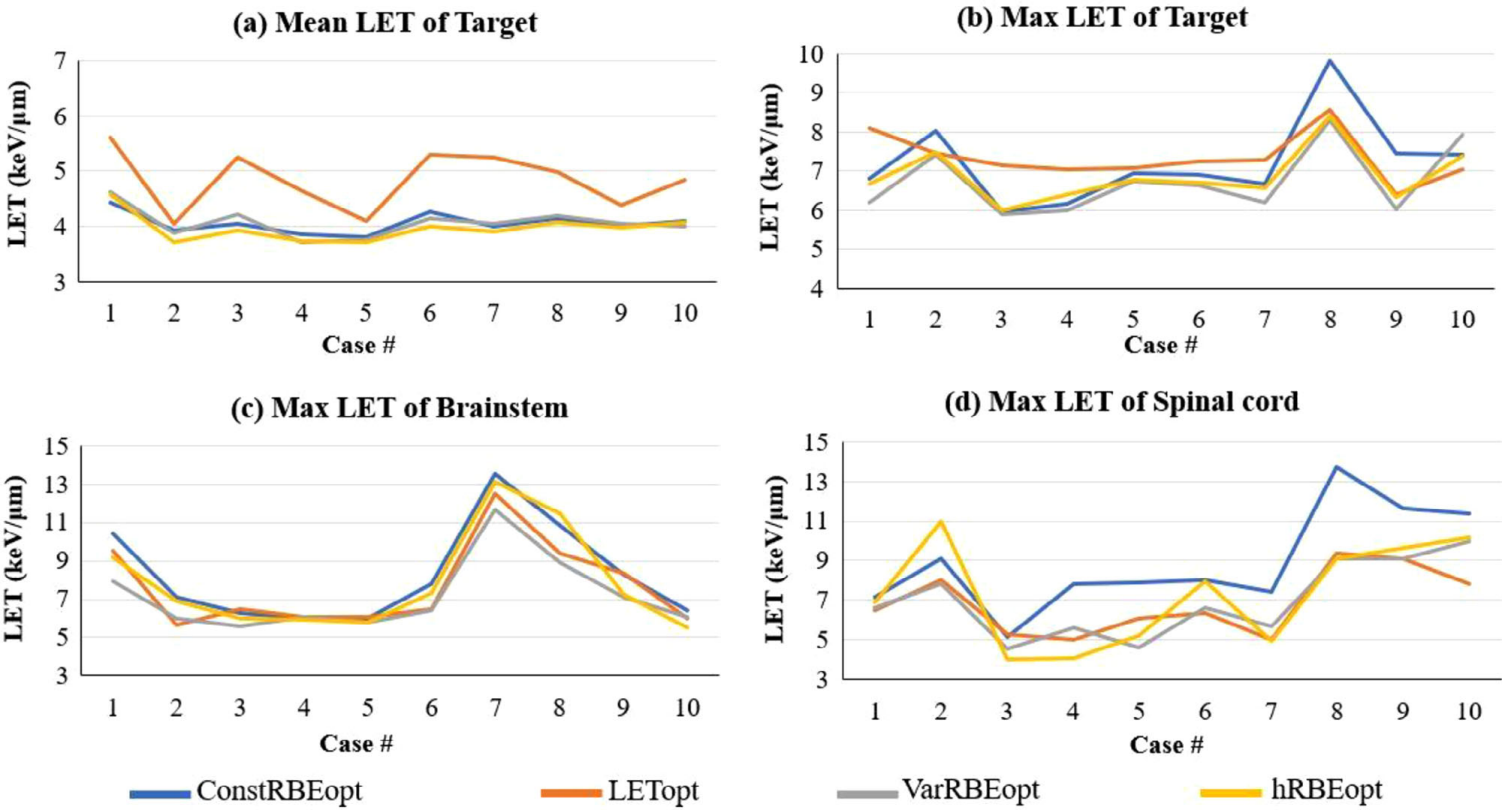
Mean (a) and max (b) LET for target, max LET for brainstem (c) and spinal cord (d) from ConstRBEopt, LETopt, VarRBEopt, and hRBEopt plans for 10 ependymoma cases.

The mean and max LET of the target and max LET of the brainstem and spinal cord obtained from each plan are shown in Figure 1, respectively. On average, LETopt led to a marked increase of mean LET in the target by 19%, 19%, and 22% compared to ConstRBEopt, VarRBEopt, and hRBEopt, respectively (*p* < 0.001 for all), for the 10 ependymoma patients. However, there was no statistically significant difference in max LET in the brainstem between LETopt and VarRBEopt or hRBEopt (*p* > 0.05 for both). For the spinal cord, reductions in max LET by LETopt and VarRBEopt compared to ConstRBEopt were statistically significant (*p* < 0.001 for both). Meanwhile, no statistically significant difference was found between LETopt, VarRBEopt and hRBEopt plans in terms of spinal cord max LET (*p* > 0.05 for all). Detailed values of LET for all plans are listed in Appendix (Tables A1, A2, A3).

**FIGURE 2:**
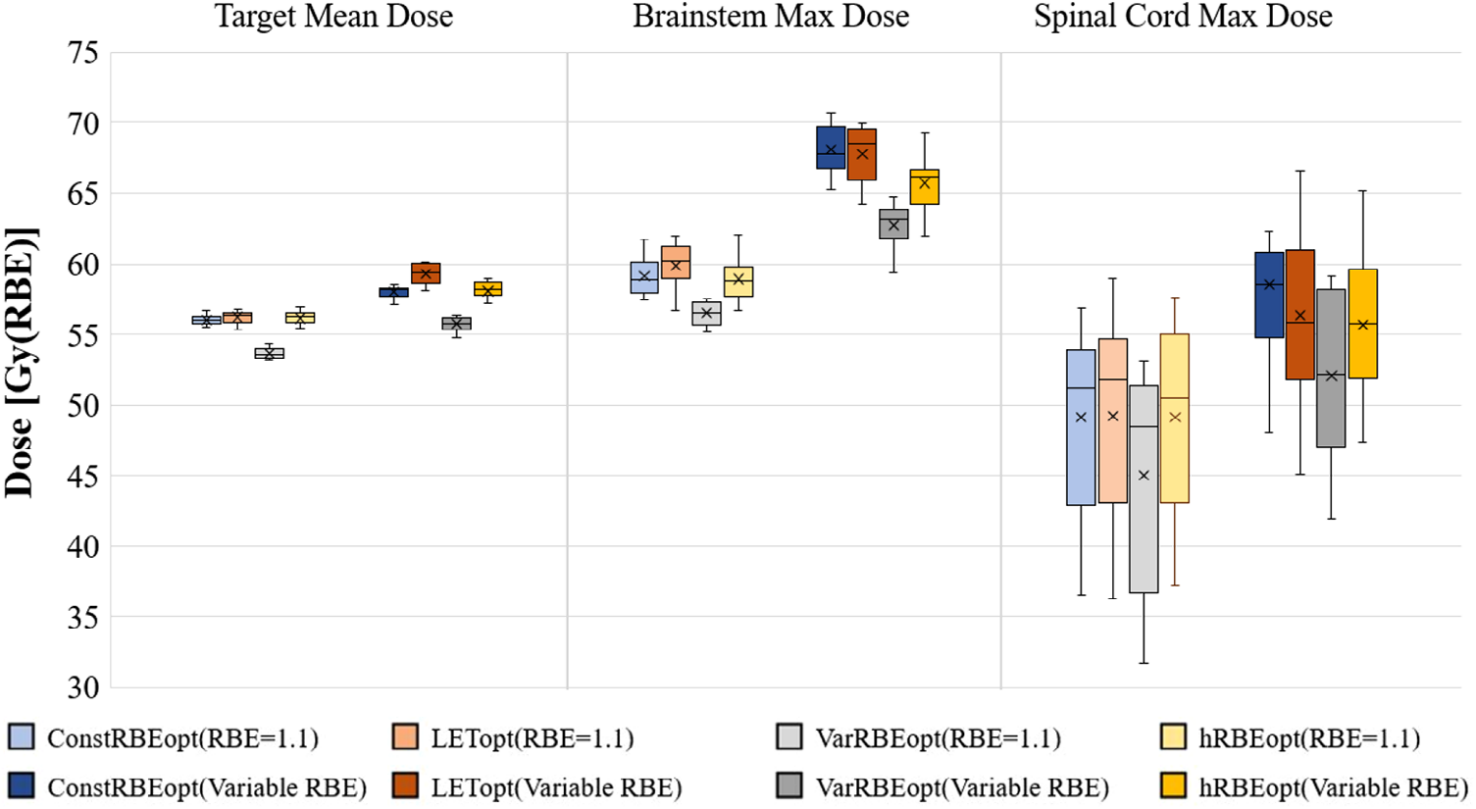
Box plot of mean dose in target, max dose in brainstem, and max dose in spinal cord of ConstRBEopt, LETopt, VarRBEopt, and hRBEopt plans recalculated with constant and variable RBE‐weighted doses for 10 ependymoma cases. All plans were normalized to have the same target coverage for the ConstRBEopt, LETopt, hRBEopt plans in terms of 1.1 RBE and the VarRBEopt plans in terms of variable RBE.

Figure 2 summarizes the target mean dose, brainstem max dose, and spinal cord max dose (maximum dose of all voxels in spinal cord) based on both constant and variable RBE for the four plans for all 10 cases. Data points for each of the cases can are included in Appendix (Tables A4, A5, A6, A7, A8, A9). LETopt increased mean variable RBE dose in the target by the most among the four optimization approaches (indicating the impact of increased LET on variable RBE‐weighted dose). The VarRBEopt plans achieved the lowest max dose in the brainstem and spinal cord among the plans with either constant or variable RBE. With constant RBE, the average of mean doses in the brainstem obtained from VarRBEopt plan was significantly lower than the ConstRBEopt, LETopt, and hRBEopt plans, respectively (*p* < 0.01 for all); but no significant difference for the spinal cord (*p* > 0.05 for all). With variable RBE, there were no significant difference in the brainstem and spinal cord mean doses either (*p* > 0.05 for all). The hRBEopt plans resulted in similar max dose in the brainstem and spinal cord compared to the ConstRBEopt plan and the LETopt plan with constant RBE (*p* > 0.05 for all, except hRBEopt vs. LETopt for brainstem); however, it outperformed those two plans with variable RBE (*p* < 0.05 for all, except hRBEopt vs. LETopt for spinal cord). Also, large variations of max doses in the spinal cord were observed among the 10 patient plans. As the VarRBEopt plans were optimized to achieve required target coverage according to variable RBE, it appears that their target coverage with RBE of 1.1, for instance target D_90%_ with a mean of 51 Gy(RBE) and ranging from 49.9 to 52.1 Gy(RBE), was lower than that of the other three plans (mean of 54 Gy(RBE)).

**FIGURE 3:**
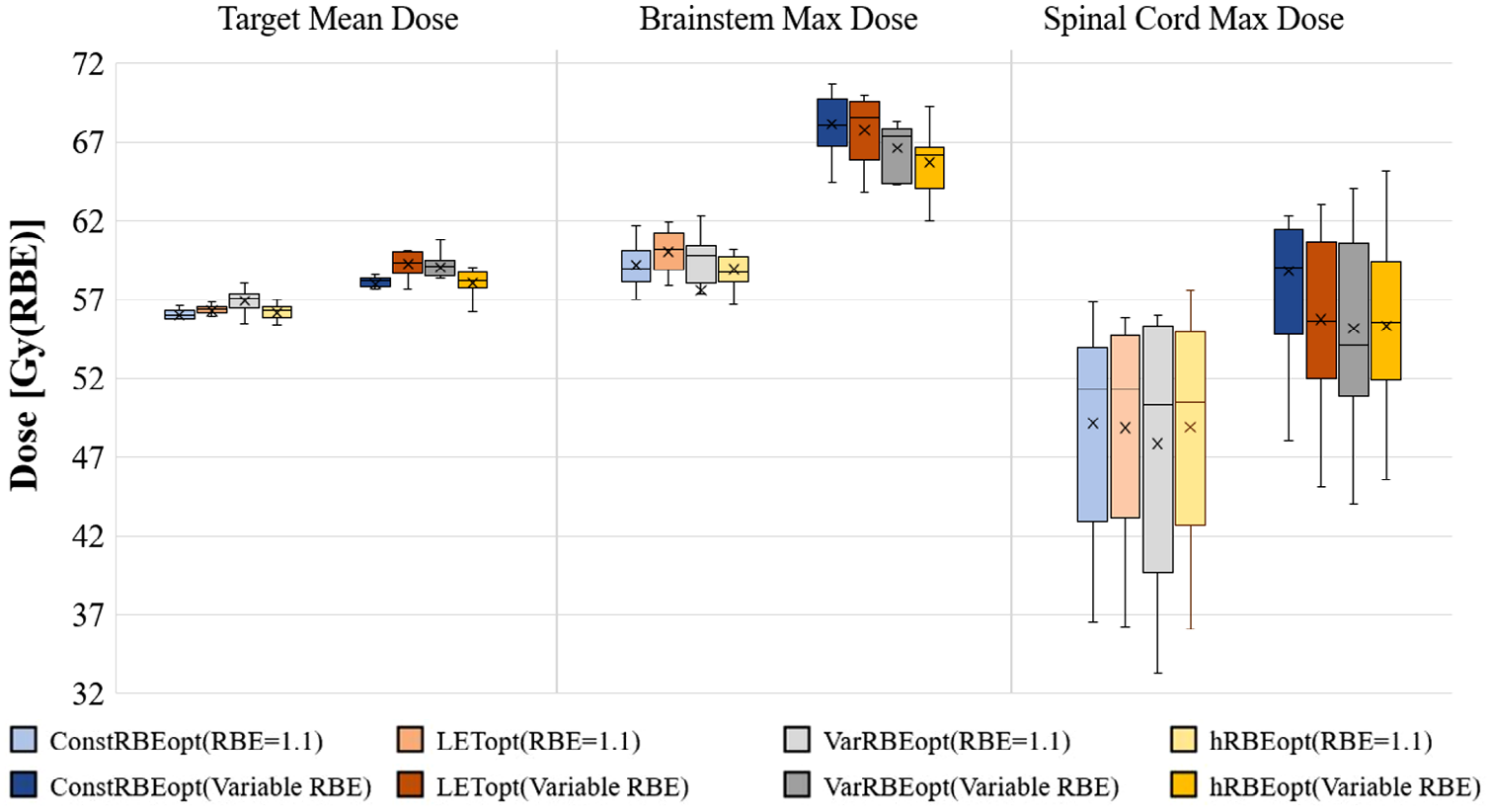
Box plot of mean dose in target, max dose in brainstem, and max dose in spinal cord of ConstRBEopt, LETopt, VarRBEopt, and hRBEopt plans recalculated with constant and variable RBE‐weighted doses for 10 ependymoma cases. All plans were normalized to have the same target coverage in terms of 1.1 RBE.

When considering the normalization of all plans to achieve the same target coverage in 1.1 RBE, as illustrated in Figure 3, the hRBEopt plans exhibited comparable target doses but displayed a significant reduction in brainstem doses compared to the ConstRBEopt plans in terms of variable RBE.

We also calculated differential EUDs, (6) and (7), based on variable RBE weighted dose for each of the biologically optimized plans compared to the ConstRBEopt plan (see Appendix—Table 10). Note that positive values of ΔEUD indicate superiority of the biologically optimized plans compared to the ConstRBEopt plan and vice versa. The average [range] composite gain of LETopt, VarRBEopt, and hRBEopt with variable RBE‐weighted dose over ConstRBEopt was 2.5 [0.08, 6.51] Gy(RBE), 4.3 [0.07, 9.89] Gy(RBE), and 2.7 [0.18, 6.26] Gy(RBE), respectively, among the 10 ependymoma cases.

**FIGURE 4:**
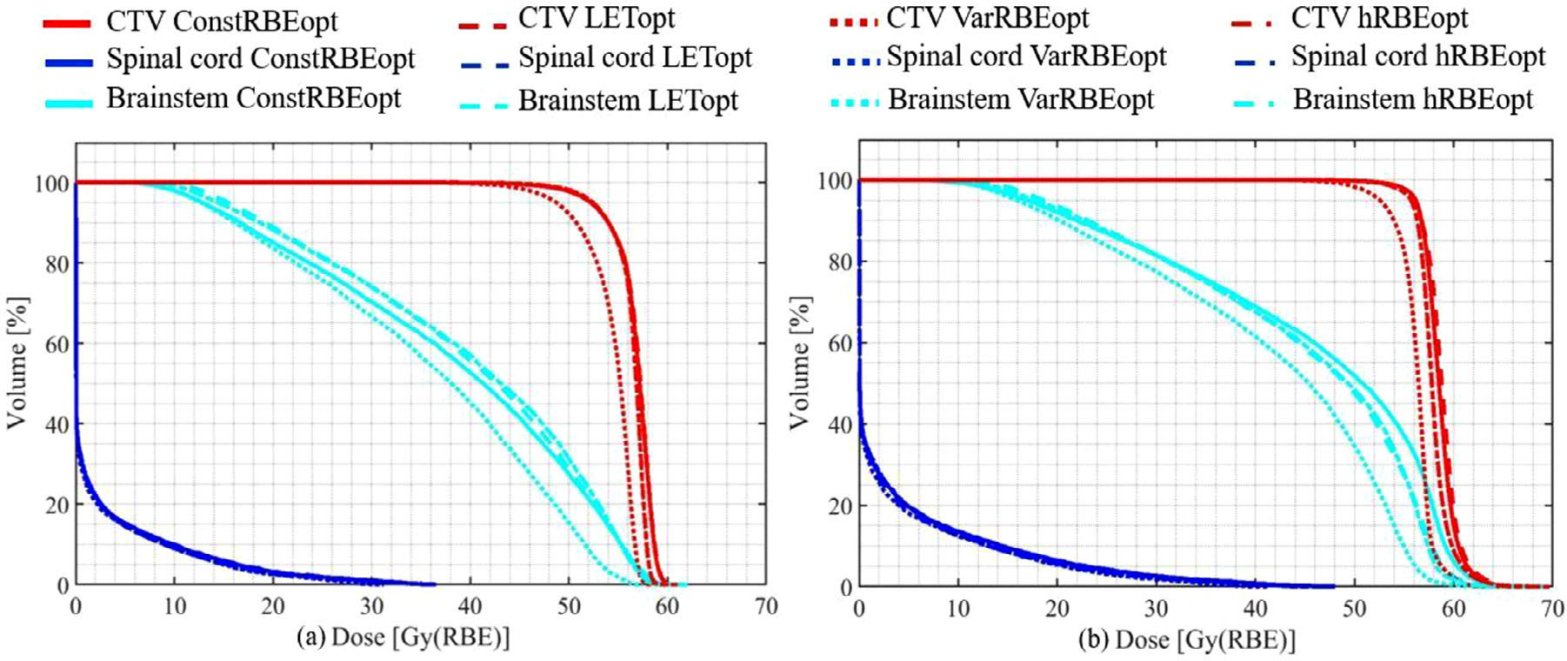
DVHs based of (a) constant and (b) variable RBE for four differently optimized plans for an example ependymoma case (case #2).

Figure 4 shows DVHs of all plans based on constant and variable RBE‐weighted doses for an example of the 10 ependymoma cases (case #2). In both scenarios, brainstem was better spared, especially in the high‐dose region, in the VarRBEopt plan than in the ConstRBEopt, LETopt, and hRBEopt plans. There was no marked difference in spinal cord DVHs among the plans. As shown in the constant RBE DVHs (Figure 4a), the D_90%_ was near 51 Gy(RBE) for the VarRBEopt plan, while all other plans had the same D_90%_ of 54 Gy(RBE).

**FIGURE 5:**
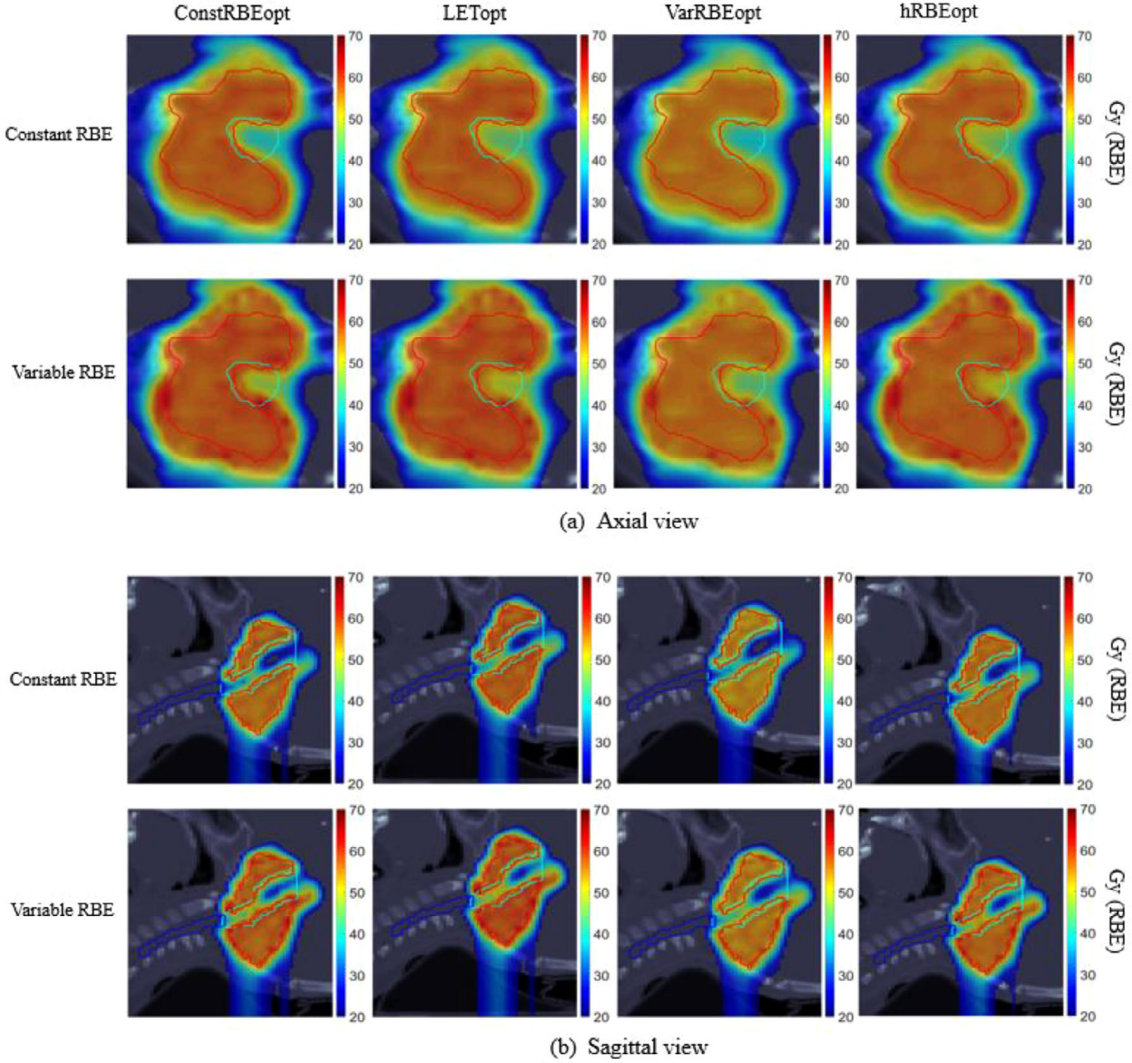
Axial and sagittal views of dose distributions obtained from the ConstRBEopt, LETopt, VarRBEopt, and hRBEopt plans for an example ependymoma case (case #2). Red contours are CTV, cyan contours are brainstem, and dark blue contours are spinal cord. Color bars represent doses in Gy(RBE).

Dose distributions in both constant and variable RBE of all four plans have been reviewed. While dose within the brainstem increased when changing from constant to variable RBE for all plans, the increase was most significant for the ConstRBEopt plan (example case #2 shown in Figure 5).

**FIGURE 6:**
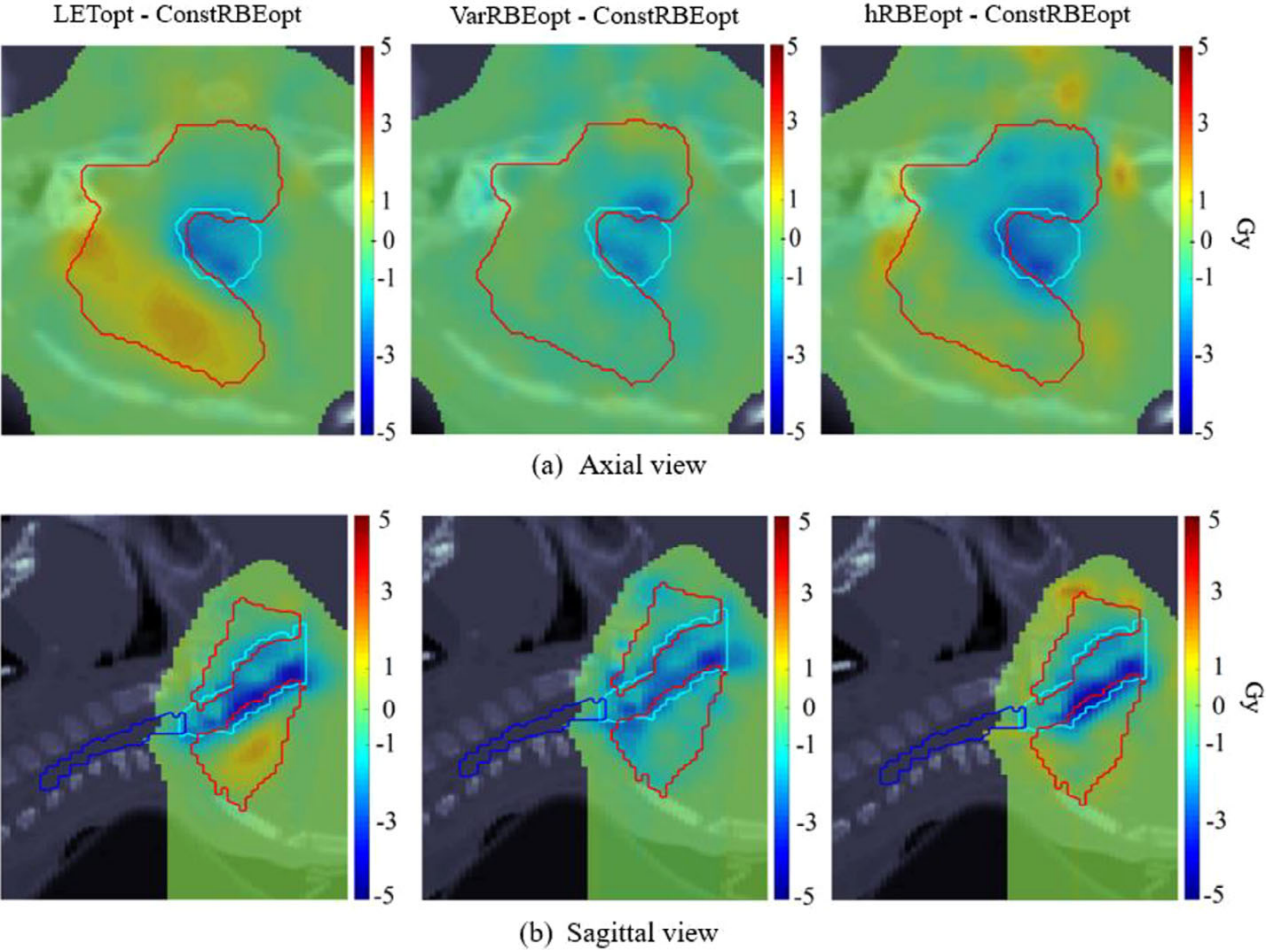
Axial and sagittal views of distribution showing difference in c LET ×  D (c = 0.04 μm/keV) obtained from the LETopt, VarRBEopt, and hRBEopt plans compared to the ConstRBEopt plan for an example ependymoma case (case #2). Red contours are CTV, cyan contours are brainstem, and dark blue contours are spinal cord. Color bars represent doses in Gy.

When evaluating the LET‐weighted dose (c LET × D), each of the three biologically optimized plans showed lower LET‐weighted dose in the brainstem compared to the ConstRBEopt plan. In addition, the hRBEopt plan showed greater reduction of LET‐weighted dose in the brainstem than did the LETopt and VarRBEopt plans, although this was at the expense of a relatively cooler CTV (see example in Figure 6).

## 4 DISCUSSION

As many aspects of the clinical use of proton therapy progress rapidly, including the transition from passive scattering to scanning pencil beams in existing and new centers and reduced planning target margins thanks to uncertainty mitigation measures, the use of a constant RBE throughout the entire irradiated volume could become more problematic. Therefore, improvement in understanding spatial RBE variation is increasingly critical. Although current treatment planning and dose prescriptions are still based on constant RBE, some proton centers have begun building capacities to assess, and even optimize, biologically effective dose (for variable RBE) or surrogates for evaluation purposes in order to assist oncologists in making clinical decisions.

Research on treatment plan optimization using variable RBE models is currently limited. Variable RBE was first incorporated into IMPT plan optimization by Frese et al.^18^ using a cost function of biological effect. Recently, Hahn et al.^43^ reported another approach in which both constant and variable RBE weighted doses are included in the cost function. So far, most studies of biologically based IMPT planning have focused on LET or LET‐weighted dose optimization.^24^, ^25^, ^26^, ^27^, ^28^, ^29^, ^38^, ^44^ Our study aimed to compare the effectiveness of RBE and LET optimization approaches for tailoring RBE and LET distributions.

As found in previous studies,^17^, ^45^ we demonstrated that IMPT plan optimization using variable RBE models can obtain comparable dosimetric quality in the domain of constant RBE while reducing variable RBE‐weighted dose in critical normal structures compared to conventional constant RBE optimization. Note that these three studies (including this one) were based on three different empirical RBE models. The Frese study used the Wilkens and Oelfke model,^46^ in which the proton tissue parameter β is independent of LET. The Hahn study used the Wedenberg model,^16^ in which proton β increases with increasing LET. The McNamara model^15^ used in the present study used a similar form of the Wedenberg model, but its parameters were fitted from more comprehensive experimental cell survival data. It is likely that the differential impact between variable and constant RBE optimization depends on the RBE model chosen. However, it is reasonable to conjecture that the trend of reducing high RBE in normal structures would be consistent across models. Additional research is needed to support such insight. One example is from a recent study by Giovannini et al.,^47^ which gave a thorough comparison of three RBE models: Carabe,^48^ Wedenberg,^16^ and local effect model.^49^

Because it employs an RBE value higher than 1.1, VarRBEopt resulted in a lower physical dose in the target (about 2.5 Gy(RBE) in mean dose) and/or OARs (about 2.3 Gy(RBE) in mean dose) compared to other approaches in our study. Similar results were observed in other studies.^17^, ^45^ Thus, the VarRBEopt plans could not be approved clinically if a target dose prescription in RBE of 1.1 must be satisfied (see example in Figure 4). To remedy this scenario for immediate clinical applications of variable RBE optimization, one could use a hybrid approach (hRBEopt) to enforce target dose criteria with RBE of 1.1 while using a variable RBE model for OAR criteria. Although the hRBEopt plans compromised on OAR sparing—reduced physical and variable RBE dose, as achieved by VarRBEopt, these plans were still more advantageous than the ConstRBEopt and LETopt plans in our study. Brainstem mean and max dose reductions by the hRBEopt plans were 1.3 and 2.4 Gy(RBE) compared to the ConstRBEopt plans, and 2.0 and 2.0 Gy(RBE) compared to the LETopt plans, respectively, on average for the 10 ependymoma cases. Based on a pairwise *t*‐test with significance level 0.05, although mean dose reduction in brainstem is not statistically significant, the max dose reduction in brainstem of hRBEopt is significant compared to ConstRBEopt and LETopt with *p*‐value 0.005 and 0.04. For the spinal cord, the corresponding reductions were 0.3 and 3.2 Gy(RBE) compared to the ConstRBEopt plans, and 0.05 and 0.4 Gy(RBE) compared to the LETopt plans. Even though the average dose reduction in the spinal cord is not statistically significant when comparing hRBEopt with ConstRBEopt and LETopt, there is a significant decrease in the maximum dose in the spinal cord in hRBEopt compared to ConstRBEopt with *p*‐value of 0.01. In another example, if all plans are normalized to have the same target coverage in 1.1 RBE, as shown in Figure 3, the hRBEopt plans achieved similar dose in target but significantly reduced dose in brainstem in variable RBE compared to the ConstRBEopt plans.

It is worth noting that some of the plans appear to exceed the typical maximum dose limits for brainstem (e.g., Dmax < 57 Gy(RBE)) or spinal cord (e.g., Dmax < 50 Gy(RBE)). It is even more so for variable RBE weighted dose (Figures 2 and 3). This is mainly because the normalization of each original plans to meet the same target coverage threshold for plan comparison purposes and the effect of variable RBE. We should emphasize that, based on our data (normalized dose distributions) in this study, biologically optimized plans could consistently achieve lower RBE dose to OARs like brainstem and spinal cord, with the same target coverage, compared to conventionally optimized plans. If such biological optimization approaches were used in clinical practice, especially for challenging cases with overlap between target volume and critical organs as seen in this study, biologically optimized plans could still be superior than the conventional plans dosimetrically.

In the present study, LET optimization was effective in increasing LET_d_ in target (19% increase of mean LET_d_ in CTV on average compared to the ConstRBEopt plans) and decreasing LET in OARs (17% decrease of mean LET in brainstem) in the present study (Appendix Table A1, A2, A3). The effect of LET optimization on LET_d_ was consistently greater than that of VarRBEopt and hRBEopt. We also found that the LETopt plans increased the variable RBE‐weighted dose in CTV by 1.25 Gy(RBE) on average compared to the ConstRBEopt plans because of the increased LET_d_. However, the advantage of LETopt for enhancing biological dose in CTV may be achieved at a cost of increased physical dose in the brainstem (Figure 2). Nevertheless, the maximum biological doses in the brainstem for the LETopt plans were not higher than the ConstRBEopt plans, due to lowered brainstem LET by LETopt (Appendix—Table 2). The difference in variable RBE weighted dose between these two sets of plans was not statistically significant (*p* = 0.7).

Although it is not trivial to obtain an accurate value for parameter c to predict RBE, c LET × D could be a useful measure in biological evaluation, especially for plan comparison. In this study, the VarRBEopt and hRBEopt plans were more effective in reducing c LET × D in brainstem than was the ConstRBEopt plan for our example case (Figure 6), even though they were optimized using a more complex RBE model. It is interesting that hRBEopt resulted in lower biological dose in some CTV voxels near the brainstem compared to the ConstRBEopt plan, especially because the physical or constant RBE doses were nearly the same for the two plans (Appendix—Table 4). In evaluation and optimization of LET or LET‐weighted dose, most studies did not use a prescribed limit to these values. For example, any LET or LET‐weighted dose greater than zero were minimized in normal tissues. The most recent study by Hahn et al.^43^ suggested a threshold of 40 Gy(RBE) for LET optimization for cranial IMPT plans based on analyzing image change (after proton therapy) data.

EUD could be another useful measure for biological evaluation of IMPT plans. With increasing numbers of planning strategies and RBE models to be evaluated, EUD and differential EUD (ΔEUD) can give planners a single composite index representing differences in plan quality. In the present study, all LETopt, VarRBEopt, and hRBEopt plans were found preferable than ConstRBE plans in various degrees according to a range of positive ΔEUD (in variable RBE) values among different plans and patients (Appendix—Table 10). When evaluating ΔEUD per VOI, that is, ΔEUD_VOI_, higher EUDs of CTV were consistently achieved in the LETopt plans, compared to the ConstRBEopt plans. For the VarRBEopt plans, although EUDs of CTV were not as high as those of the ConstRBEopt plans, EUDs of brainstem and spinal cord were mostly lower than for the ConstRBEopt plans, which resulted in positive overall ΔEUDs for all the cases. For the hRBEopt plans, positive ΔEUDs were mainly attributable to lower EUDs of brainstem and spinal cord compared to the ConstRBEopt plans. In addition, with constant RBE, the ΔEUDs comparing LETopt, VarRBEopt, and hRBEopt with ConstRBEopt, respectively, were less than 1 Gy on average for all cases (Appendix—Table 11).

Despite the dosimetric advantages of the hybrid RBE optimization approach (hRBEopt) showed in our study, clinical implementation of such strategy could be controversial, as there is no clinical evidence that the RBE would be constant in tumor but variable in normal tissues in a patient. While the ultimate solution in proton planning remains using variable RBE as accurately as possible throughout the patient's body, the hybrid approach can provide an effective alternative to reduce variable RBE‐weighted dose in critical serial organs. Similar findings were reported in other recent studies.^43^

Regarding computational time, VarRBEopt took about 55% longer on average to solve than did ConstRBEopt because of its higher complexity in calculating the gradient of its objective function and more iterations to converge. Solving hRBEopt and LETopt took 39% and 10% more time than ConstRBEopt, respectively. All plans took less than 6 min for optimization, including time used for calculating dose and LET influence matrices.

One limitation of the present study is that robust optimization was not incorporated in the tested planning approaches. It has been suggested that robust optimization (against physical uncertainties) might reduce the impact of variable RBE.^50^, ^51^ Variable RBE optimization is particularly suitable for existing robust optimization methods developed for IMPT planning. One only needs to replace constant RBE with variable RBE in the optimization criteria. Nevertheless, incorporation of physical uncertainties into optimization of biological dose requires additional investigations. Another future step to further exploit the biological effect of protons could be beam angle optimization. It is likely that use of more—or nonintuitive—beam angles could lead to improved biological dose distribution with added degrees of freedom in optimization. A more challenging question requiring thorough study is how many more angles would be truly beneficial, as in the emerging proton arc therapy.^52^, ^53^

## 5 CONCLUSION

In our study of variable RBE and LET effect of protons in 10 anatomically challenging ependymoma cases, biologically based optimization approaches consistently outperformed standard optimization using a constant RBE for IMPT treatment planning. While directly optimizing variable RBE‐weighted dose can achieve substantial benefit in sparing critical organs like the brainstem compared to constant RBE or LET optimization approaches, it may lead to target underdosing with the current standard of 1.1 RBE. With a hybrid approach of assuming constant RBE for target and variable RBE for normal tissues, the benefit of variable RBE optimization for brainstem protection can still exceed that of other approaches including LET optimization.

## AUTHOR CONTRIBUTIONS

Hadis Moazami Goudarzi and Wenhua Cao developed the study and wrote the manuscript. Hadis Moazami Goudarzi implemented the methods and performed data collection and analysis. Gino Lim, David Grosshans, and Radhe Mohan provided critical technical and clinical advices, and helped revising the manuscript. All authors gave major contributions to this study and approved the submitted manuscript.

## CONFLICT OF INTEREST STATEMENT

The authors have no conflicts of interest to disclose.

## 1

Tables A1, A2, A3 list the mean and max LET_d_ values in target and OARs from ConstRBEopt, LETopt, VarRBEopt, and hRBEopt plans for all cases.

**TABLE A1:** LET_d_ (keV/um) values for target.

Case	ConstRBEopt		LETopt		VarRBEopt		hRBEopt	
	Mean	Max	Mean	Max	Mean	Max	Mean	Max
1	4.44	6.81	5.62	8.11	4.62	6.20	4.58	6.69
2	3.92	8.03	4.06	7.46	3.89	7.42	3.71	7.50
3	4.05	5.96	5.26	7.14	4.22	5.91	3.93	5.98
4	3.87	6.15	4.65	7.06	3.72	5.98	3.74	6.39
5	3.82	6.96	4.11	7.09	3.77	6.75	3.72	6.78
6	4.27	6.90	5.30	7.25	4.15	6.65	4.00	6.71
7	4.01	6.66	5.26	7.29	4.05	6.20	3.91	6.57
8	4.15	9.83	4.98	8.56	4.19	8.31	4.07	8.41
9	4.01	7.44	4.39	6.40	4.04	6.04	3.97	6.33
10	4.10	7.41	4.85	7.06	4.00	7.93	4.07	7.37

**TABLE A2:** LET_d_ (keV/um) values for brainstem.

Case	ConstRBEopt		LETopt		VarRBEopt		hRBEopt	
	Mean	Max	Mean	Max	Mean	Max	Mean	Max
1	4.34	10.40	3.48	9.51	3.79	7.90	4.09	9.17
2	4.45	7.13	3.24	5.64	3.69	5.97	3.12	6.87
3	2.64	6.28	2.30	6.48	2.34	5.61	2.02	6.02
4	3.59	6.02	2.99	6.07	3.25	6.08	3.03	5.92
5	1.91	6.00	1.83	6.05	1.81	5.79	1.71	5.76
6	3.49	7.78	2.72	6.51	2.98	6.42	2.32	7.34
7	3.42	13.54	2.51	12.51	3.13	11.65	3.02	13.09
8	2.69	10.83	2.52	9.35	2.52	8.93	2.39	11.49
9	2.03	8.27	1.73	8.33	2.05	7.10	1.98	7.27
10	2.61	6.42	2.62	5.98	2.63	6.05	2.55	5.52

**TABLE A3:** LET_d_ (keV/um) values for spinal cord.

Case	ConstRBEopt		LETopt		VarRBEopt		hRBEopt	
	Mean	Max	Mean	Max	Mean	Max	Mean	Max
1	2.64	7.15	2.19	6.52	2.26	6.65	2.62	6.92
2	0.94	9.10	0.72	8.05	0.80	7.82	0.91	10.96
3	0.26	5.16	0.24	5.32	0.23	4.55	0.21	4.04
4	0.58	7.82	0.40	5.02	0.42	5.60	0.29	4.06
5	2.46	7.87	1.71	6.12	1.53	4.64	1.39	5.25
6	0.41	8.05	0.33	6.35	0.34	6.62	0.37	7.98
7	1.95	7.45	1.29	5.01	1.53	5.67	1.17	4.97
8	4.10	13.73	2.70	9.38	2.87	9.07	2.63	9.10
9	0.93	11.63	0.72	9.13	0.81	9.10	0.76	9.66
10	2.05	11.38	1.78	7.84	1.46	9.98	1.46	10.18

Tables A4, A5, A6 summarize constant RBE‐weighted dose for all cases.

**TABLE A4:** Mean dose (Gy(RBE)) in target based on constant RBE‐weighted dose.

Case	ConstRBEopt	LETopt	VarRBEopt	hRBEopt
1	55.79	56.27	53.55	56.12
2	56.66	56.71	54.34	56.31
3	56.27	56.43	53.99	56.02
4	55.66	55.71	53.47	54.89
5	56.1	56.22	54	56.48
6	55.87	56.43	53.28	56.98
7	55.51	55.94	53.34	55.42
8	56.02	55.37	53.6	56.62
9	56.32	56.81	53.77	56.47
10	56.07	56.37	53.20	56.25

**TABLE A5:** Mean and max dose (Gy(RBE)) in brainstem based on constant RBE‐weighted dose.

Case	ConstRBEopt		LETopt		VarRBEopt		hRBEopt	
	Mean	Max	Mean	Max	Mean	Max	Mean	Max
1	27.08	58.18	27.8	60	22.87	57.53	27.22	58.54
2	38.43	60.64	39.75	61.8	35.81	57.56	39.89	62.09
3	24.87	58.49	25.58	59.18	27.32	56.52	28.54	57.99
4	43.48	57.74	46.77	58.32	43.42	55.31	44.32	56.69
5	27.85	61.72	27.75	60.38	24.94	57.2	24.45	58.96
6	25.07	59.3	28.6	61.03	24.09	55.76	22.94	60.16
7	17.9	57.41	23.84	58.09	17.49	55.18	19.79	56.95
8	22.18	59.81	22.44	60.46	19.22	56.56	20.17	59.59
9	20.36	59.97	19.95	61.96	18.65	57.07	19.50	59.71
10	31.41	58.07	32.19	59.30	29.11	56.27	31.4	58.54

**TABLE A6:** Mean and max dose (Gy(RBE)) in spinal cord based on constant RBE‐weighted dose.

Case	ConstRBEopt		LETopt		VarRBEopt		hRBEopt	
	Mean	Max	Mean	Max	Mean	Max	Mean	Max
1	8.48	43.66	8.1	44.17	6.54	37.19	8.33	44.07
2	2.55	36.56	2.55	36.28	2.30	31.26	2.42	36.08
3	1.58	53.53	1.69	53.95	1.71	53.07	1.72	54.98
4	2.26	52.78	2.33	52.4	2.24	48.78	2.17	50.58
5	12.54	55.24	13.16	54.45	14.27	53.07	14.15	54.94
6	0.94	40.57	0.94	40.01	0.84	35.21	0.83	38.46
7	6.16	49.71	5.24	45.44	5.17	42.82	5.72	47.15
8	9.34	56.87	10.71	55.37	9.16	49.89	11.8	57.61
9	2.78	52.78	3.59	55.84	3.33	51.07	3.36	54.66
10	6.08	49.63	7.14	50.56	5.20	47.19	5.69	50.42

Tables A7, A8, A9 summarize variable RBE‐weighted dose for all cases.

**TABLE A7:** Mean dose (Gy(RBE)) in target based on variable RBE‐weighted dose.

Case	ConstRBEopt	LETopt	VarRBEopt	hRBEopt
1	58.15	60.03	56.1	58.72
2	58.55	58.77	56.16	57.93
3	58.46	60.13	56.33	58.07
4	57.15	58.11	54.78	56.24
5	57.85	58.34	55.66	58.11
6	58.18	60.04	55.4	59
7	57.37	59.31	55.22	57.18
8	58.28	59.6	55.87	58.81
9	58.17	59.11	55.59	58.25
10	58.24	59.47	56.38	58.38

**TABLE A8:** Mean and max dose (Gy(RBE)) in brainstem based on variable RBE‐weighted dose.

Case	ConstRBEopt		LETopt		VarRBEopt		hRBEopt	
	Mean	Max	Mean	Max	Mean	Max	Mean	Max
1	32.76	68.78	32.13	65.98	27.43	59.41	32.64	67.11
2	45.43	67.43	44.47	68.48	41.21	63.77	44.39	69.26
3	28.93	67.84	28.95	69.70	30.86	64.03	31.54	64.41
4	47.63	65.27	49.44	64.24	46.97	62.08	47.46	63.62
5	31.14	70.68	30.85	69.95	27.87	64.72	27.21	66.38
6	30.23	66.75	32.67	69.33	28.31	62.9	25.96	62.02
7	22.56	67.75	27.35	66.1	21.69	63.36	23.97	66.5
8	26.12	70.7	26.14	68.61	22.73	62.25	20.29	66.31
9	22.75	69.4	21.82	65.62	20.99	61.1	21.79	65.44
10	35.7	66.72	36.39	69.45	33.02	63.58	35.4	66.06

**TABLE A9:** Mean and max dose (Gy(RBE)) in spinal cord based on variable RBE‐weighted dose.

Case	ConstRBEopt		LETopt		VarRBEopt		hRBEopt	
	Mean	Max	Mean	Max	Mean	Max	Mean	Max
1	11.88	54.59	10.86	53.84	9.29	47.15	11.79	55.67
2	3.78	48.06	3.51	45.08	3.31	41.42	3.57	45.55
3	1.95	59.17	2.04	60.35	2.04	57.00	2.02	59.09
4	3.03	56.49	2.88	55.79	2.81	52.3	2.53	52.09
5	15.87	60.31	15.47	55.84	16.26	54.92	15.96	56.08
6	1.42	54.87	1.34	51.95	1.22	46.87	1.25	51.66
7	8.57	58.28	6.81	51.47	6.99	49.66	7.09	51.97
8	14.63	72.72	14.45	63.07	12.88	58.8	14.99	65.17
9	4.09	62.32	4.61	61.61	4.46	59.1	4.44	60.46
10	8.85	58.85	9.72	58.40	7.01	50.89	7.6	55.52

**TABLE A10:** ΔEUD_VOI_ and ΔEUD (in Gy(RBE)) based on variable RBE × Dose for 10 ependymoma cases.

Case #	ΔEUD LETopt − ConstRBEopt				ΔEUD VarRBEopt − ConstRBEopt				ΔEUD hRBEopt − ConstRBEopt			
	CTV	BS	Cord	Total	CTV	BS	Cord	Total	CTV	BS	Cord	Total
**1**	2.21	–1.03	–1.17	**4.41**	–1.91	–6.22	–5.58	**9.89**	1.04	0.24	0.62	**0.18**
**2**	0.18	–1.82	–1.33	**3.33**	–2.57	–4.76	–3.71	**5.9**	–0.57	–1.68	–1.23	**2.34**
**3**	1.57	–0.01	1.12	**0.46**	–2.13	–2.02	–0.18	**0.07**	–0.4	–1.05	0.28	**0.37**
**4**	0.89	0.2	–0.55	**1.24**	–2.33	–1.94	–2.27	**1.88**	–0.91	–1.53	–2.62	**3.24**
**5**	0.49	–0.63	–1.71	**2.83**	–2.15	–4.02	–1.01	**2.88**	0.28	–4.48	–1.5	**6.26**
**6**	1.7	1.14	–1.36	**1.92**	–2.88	–1.68	–3.92	**2.72**	0.49	–2.01	–1.67	**4.17**
**7**	1.78	0	–4.73	**6.51**	–2.22	–3.37	–5.64	**6.79**	–0.2	1.07	–3.86	**2.59**
**8**	1.18	–0.14	–2.85	**4.17**	–3	–5.56	–6.21	**8.77**	0.54	–3.27	–1.42	**5.23**
**9**	0.8	–0.43	1.15	**0.08**	–2.59	–2.55	–0.23	**0.19**	0.09	–0.55	0.24	**0.4**
**10**	1.13	0.39	0.6	**0.14**	–3.05	–2.81	–4.54	**4.3**	0.11	–0.31	–1.89	**2.31**
**Mean**	1.193	–0.233	–1.083	**2.509**	–2.483	–3.493	–3.329	**4.339**	0.047	–1.357	–1.305	**2.709**

**TABLE A11:** ΔEUD based on constant RBE‐weighted dose for all cases.

Case #	ΔEUD LETopt ‐ ConstRBEopt				ΔEUD VarRBEopt ‐ ConstRBEopt				ΔEUD hRBEopt ‐ ConstRBEopt			
	CTV	BS	Cord	Total	CTV	BS	Cord	Total	CTV	BS	Cord	Total
1	0.56	–0.08	–0.4	**1.04**	–3.31	–5.45	–5.06	**7.2**	0.34	–0.03	–0.07	**0.44**
2	0.18	0.58	0.28	**–0.68**	–2.73	–3.15	–2.81	**3.23**	–0.01	1.09	–0.26	**–0.84**
3	0.01	–0.03	0.7	**–0.66**	–2.48	–1.61	0.09	**–0.96**	–0.19	0.32	1.17	**–1.68**
4	0.01	0.97	0.57	**–1.53**	–2.23	–1.52	–1.21	**0.5**	–0.73	–0.54	–0.33	**0.14**
5	0.06	–0.28	–0.17	**0.51**	–2.47	–3.36	0.88	**0.01**	0.25	–3.53	0.95	**2.83**
6	0.27	1.94	–0.43	**–1.24**	–3.36	1.79	–2.68	**–2.47**	0.46	3.39	–1.14	**–1.79**
7	0.21	1.99	–2.93	**1.15**	–2.61	–2.66	–4.46	**4.51**	–0.09	4.02	–1.43	**–2.68**
8	–0.02	0.04	1.23	**–1.29**	–7.38	–4.71	–1.81	**–0.86**	0.83	–2.65	2.51	**0.97**
9	0.45	0.34	3.75	**–3.64**	–2.66	–2.35	1.3	**–1.61**	0.29	–0.2	2.43	**–1.94**
10	0.19	0.01	1.02	**–0.84**	–1.91	–1.18	–1.52	**0.79**	0.09	0.07	0.14	**–0.12**
**Mean**	0.192	0.548	0.362	**–0.718**	–3.114	–2.42	–1.728	**1.034**	0.095	0.214	0.154	**–0.273**
